# Ontogeny specification and epigenetic regulation of macrophage plasticity

**DOI:** 10.3389/fimmu.2025.1676953

**Published:** 2025-09-23

**Authors:** Han-Ying Huang, Xin-Nan Zheng, Lin Tian

**Affiliations:** State Key Laboratory of Oncology in South China, Guangdong Provincial Clinical Research Center for Cancer, Sun Yat-sen University Cancer Center, Guangzhou, China

**Keywords:** macrophage plasticity, epigenetic reprogram, ontogeny, niche factor, *In situ* profiling system

## Abstract

Macrophages are ubiquitously distributed across tissues, playing pivotal roles in maintaining homeostasis under physiological conditions and modulating disease progression in pathological contexts. Although the classic M1/M2 classification of macrophage polarization provides a useful framework, it significantly oversimplifies the plasticity and heterogeneity of these cells. Recent advances that combine lineage tracing with multi-omic profiling have unveiled new insights into macrophage functional specification. In this mini-review, we examine how ontogeny, environmental cues, and genetic as well as epigenetic factors converge to drive macrophage plasticity through epigenetic reprogramming. Additionally, we highlight cutting-edge *in situ* profiling techniques that facilitate the study of macrophages within their native tissue microenvironment. A deeper understanding of macrophage plasticity promises to elucidate fundamental regulatory mechanisms and uncover novel therapeutic targets, paving the way for transformative disease treatments.

## Introduction

Macrophages stand out among mammalian cells for their extraordinary functional and phenotypic diversity, reflected in their specialized identities across different tissues (1–3). In the brain, these immune cells are termed *microglia* due to their glial-like morphology (4), while in the liver they are known as Kupffer cells—named after their discoverer, Karl Wilhelm von Kupffer (5). Bone-resident macrophages (osteoclasts) similarly exhibit unique specialization for bone resorption (6). Despite their tissue-specific adaptations, all macrophages share core characteristics: expression of markers like CD68, IBA-1, and F4/80 (mouse-specific), and phagocytic activity critical for maintaining tissue homeostasis.

Beyond their functional versatility, macrophages are unique in their developmental origins. Unlike most immune cells, they can arise from embryonic progenitors independent of bone marrow hematopoiesis (7, 8). These embryonic-derived populations self-renew with minimal contribution from circulating monocytes under steady-state conditions. However, during injury or inflammation, monocytes infiltrate tissues and differentiate into functional macrophages—a plasticity rigorously demonstrated through murine lineage-tracing studies (9). Remarkably, macrophages retain plasticity even after terminal differentiation, as demonstrated by their ability to undergo environmental reprogramming. Striking evidence reveals that intratracheally administered macrophages can acquire alveolar macrophage-like characteristics upon lung engraftment (10), underscoring their extraordinary capacity to adapt to new tissue microenvironments.

The M1/M2 macrophage definition - developed through *in vitro* cytokine treatments to model distinct differentiation pathways - has served as a foundational framework for understanding macrophage polarization (11). However, this binary classification fails to capture the complexity of *in vivo* conditions, where dynamic cell-cell interactions and multifaceted microenvironmental cues converge (12). In this mini-review, we explore three critical dimensions: (1) the coordinated regulation of macrophage specification by ontogenetic and environmental factors, (2) the genetic and epigenetic mechanisms governing their plasticity, and (3) innovative *in situ* tools enabling their study in native contexts. By synthesizing these perspectives, we seek to stimulate new research directions in macrophage biology and advance therapeutic development.

## Macrophage specification through nature and nurture factors

The relative contributions of ontogeny (“nature”) versus environmental cues (“nurture”) to macrophage specification have long been debated, with evidence supporting both perspectives (13, 14). Environmental dominance is exemplified by studies showing that mouse peritoneal macrophages transplanted into alveolar air spaces upregulate alveolar macrophage-specific genes. Similarly, in Kupffer-cell-ablated livers, repopulating monocyte-derived macrophages adopt a gene expression profile largely convergent with resident Kupffer cells (15, 16). However, these monocyte-derived cells fail to express Timd4, a conserved Kupffer cell identity marker, revealing ontogenetic constraints (17). Likewise, hematopoietic stem cell-derived progenitors that repopulate the brain after microglia depletion do not express Sall1, even after extended periods, further underscoring the indispensable role of lineage-specific factors absent in bone marrow-derived precursors (18–20).

The key to distinguish the ontogeny effect from the environmental signals is to identify cis-acting regulatory DNA sequences, which can be either proximal promoter elements or more distal enhancers, and trans-acting regulatory proteins, which can be a signal transduction protein in response to environmental cues.

Given that macrophages from different mouse strains exhibit distinct polarization patterns, researchers have leveraged strain-specific single nucleotide polymorphisms (SNPs) as a form of *in vivo* mutagenesis screening (13). Notably, PU.1 and C/EBPα—two lineage-determining transcription factors (LDTFs) in macrophages—display strain-specific binding patterns at regulatory regions. These differential binding events correlate with altered expression of nearby genes, implicating cis-regulatory elements in shaping macrophage phenotypes. With the advent of CRISPR-based genome editing, it is now feasible to directly interrogate the functional role of these cis-regulatory elements by deleting or modifying transcription factor binding motifs. Strikingly, deletion of a super-enhancer that interacts with the promoter of Sall1 (a microglia-specific LDTF) abolishes Sall1 expression specifically in microglia and severely impairs their responsiveness to the TGFβ–SMAD signaling axis (21).

The significance of environmental or niche factors extends beyond acute depletion models of resident macrophages to inflammatory diseases like hepatitis and cancer. While inflammatory signals primarily recruit blood monocytes, they also induce profound epigenetic reprogramming in macrophages, driving their phenotypic and functional plasticity. Notably, these environmental trans-regulatory proteins act in concert with LDTFs. For instance, in metabolic dysfunction-associated steatohepatitis, the nuclear receptor LXRα (encoded by the Kupffer cell identity gene Nr1h3) cooperates with ATF3 to upregulate Trem2 and Cd9 expression, shifting Kupffer cells toward a monocyte-derived macrophage-like state (22).

**Figure 1:**
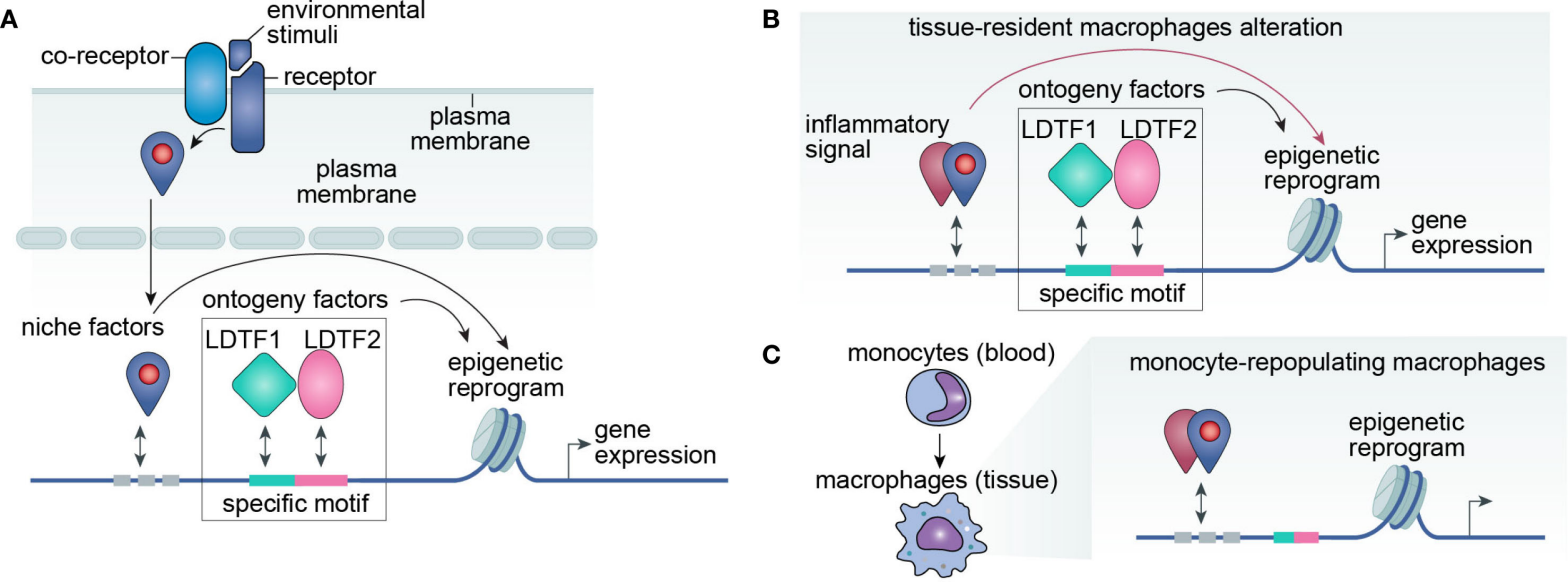
LDTF-niche factor interplay drives macrophage plasticity through epigenetic regulation. **(A)** Conceptual framework. Macrophage phenotype is primarily determined by its transcriptome, which emerges from the integrated effects of ontogenetic programming and environmental signals. Ontogenetic factors include lineage-determining transcription factors (LDTFs) and their cognate DNA regulatory elements, while environmental inputs comprise signaling cascades that cooperate with LDTFs to modulate gene expression. Identification of key LDTFs, their genomic binding motifs, and interacting co-factors will provide critical insights into the molecular mechanisms governing macrophage plasticity. **(B, C)** Niche-mediated reprogramming mechanisms. Environmental cues modulate macrophage function through two distinct pathways: **(B)** Direct epigenetic reprogramming of tissue-resident macrophages via niche-derived signaling molecules, or **(C)** Recruitment and differentiation of monocyte-derived inflammatory macrophages that primarily respond to niche signals rather than ontogenetic impriting.

Critically, neither ontogeny nor environmental cues operate in isolation. Instead, their integration enables macrophages to maintain tissue homeostasis while adapting to external stimuli (**Figure 1**). LDTFs serve as pivotal mediators that bridge ontogenetic programming and environmental responses, thereby defining macrophage functional specialization. Identifying context-specific LDTFs in physiological and pathological settings is essential. Moreover, mapping their DNA binding sites (cis-regulatory elements) and interacting nuclear partners (trans-regulatory proteins) will provide mechanistic insights into this dynamic process.

## Genetic and epigenetic factors drive macrophage plasticity

Tumors primarily accumulate mutations in malignant cells to sustain uncontrolled growth, but somatic mutations also occur in non-malignant cells, particularly in age-related conditions. When these mutations arise in hematopoietic stem and progenitor cells (HSPCs), they result in Clonal Hematopoiesis of Indeterminate Potential (CHIP) (23). Notably, about 75% of CHIP variants affect one of three epigenetic regulators: DNMT3A (involved in *de novo* DNA CpG methylation), TET2 (a key mediator of DNA demethylation and histone deacetylase recruitment), and ASXL1 (a component of the Polycomb Repressive Complex 2 that facilitates H3K27 trimethylation). CHIP has been associated with various pathologies, including kidney injury, diabetes, cardiovascular disease, and cancer. For instance, a recent clinical study of 421 non-small cell lung cancer patients revealed that 42% harbored CHIP mutations. Intriguingly, TET2 mutations in CHIP were linked not only to increased tumor-associated macrophages but also to their enhanced immunosuppressive phenotype (24). Thus, loss-of-function mutations in these genes appear to release epigenetic repression, driving macrophage plasticity and bridging genetic alterations with epigenetic dysregulation.

Beyond CHIP-mediated plasticity, metabolic reprogramming serves as another critical regulator of macrophage functional adaptation (25). The connection between cellular metabolism and macrophage polarization is well-established, particularly in the context of M1/M2 paradigms. Reactive nitrogen and oxygen species, for instance, are known to drive M1 polarization (26). In tumor microenvironments, macrophages emerge as the predominant glucose consumers, fueling both glycolytic and tricarboxylic acid (TCA) cycle activity. Notably, TCA cycle intermediates function as key epigenetic modulators: succinate and fumarate regulate DNA methylation (by altering 5-mC/5-hmC ratios) and histone methylation (particularly at H3K9, H3K27, and H3K36), while acetyl-CoA directly modulates histone acetylation states (27). These metabolic-epigenetic intersections provide a mechanistic basis for how environmental cues can shape macrophage plasticity.

The epigenetic reprogramming—including chromatin remodeling and histone modifications—plays a pivotal role in cellular adaptability (28, 29). For gene expression to initiate, enhancer and promoter regions must first become accessible, a challenge when these regions are tightly packed as heterochromatin. Chromatin remodelers regulate nucleosome positioning and decompaction, enabling transcription factor binding. This process is closely tied to post-translational modifications of histone tails, such as acetylation, methylation, and phosphorylation. For example, H3K27ac marks transcriptionally active regions, whereas H3K27me3 promotes heterochromatin formation and gene silencing.

Our recent work reveals that the bivalent H3K27 modification (acetylation vs. trimethylation) is mutually exclusive and critically regulates the phenotypic plasticity of Kupffer cells in liver malignancies, including metastasis and hepatocellular carcinoma (30, 31). In healthy liver tissue, H3K27ac maintains the expression of Kupffer cell identity genes (Timd4, Clec4f, Id3). However, upon tumor infiltration, H3K27me3 replaces H3K27ac at these loci, while H3K27ac shifts to immunosuppressive gene enhancers (e.g., Spp1, Trem2), reprogramming Kupffer cells from anti-tumor to pro-tumor effectors. Notably, Spp1 and Trem2 exhibit open chromatin even in normal Kupffer cells, suggesting that histone modification switching serves as a rapid epigenetic reprogramming mechanism to drive functional plasticity. However, how lineage-specific factors cooperate with environmental factors to drive epigenetic reprogramming remains unclear and warrants further investigation.

## *In situ* tools for probing macrophage plasticity

Traditional studies of macrophage plasticity have relied on *in vitro* differentiation models, where bone marrow-derived cells are cultured with macrophage colony-stimulating factor (M-CSF) and other cytokines to induce macrophage polarization (32). These models have proven particularly valuable in elucidating the phenomenon of trained immunity, a process whereby innate immune cells develop enhanced responsiveness to secondary stimuli through epigenetic reprogramming (evidenced by characteristic histone modifications and chromatin accessibility alterations) and metabolic rewiring (33). While *in vitro* epigenetic profiling of cultured macrophages has provided mechanistic insights into trained immunity, these models oversimplify the complex tissue milieu, where multiple signals act in concert to shape macrophage function.

Another common approach involves enzymatic tissue dissociation, followed by macrophage labeling with fluorescent antibodies and fluorescence-activated cell sorting (FACS). However, both mechanical/enzymatic digestion and FACS impose cellular stress, potentially altering macrophage states and introducing artifacts into functional profiling.

**Figure 2:**
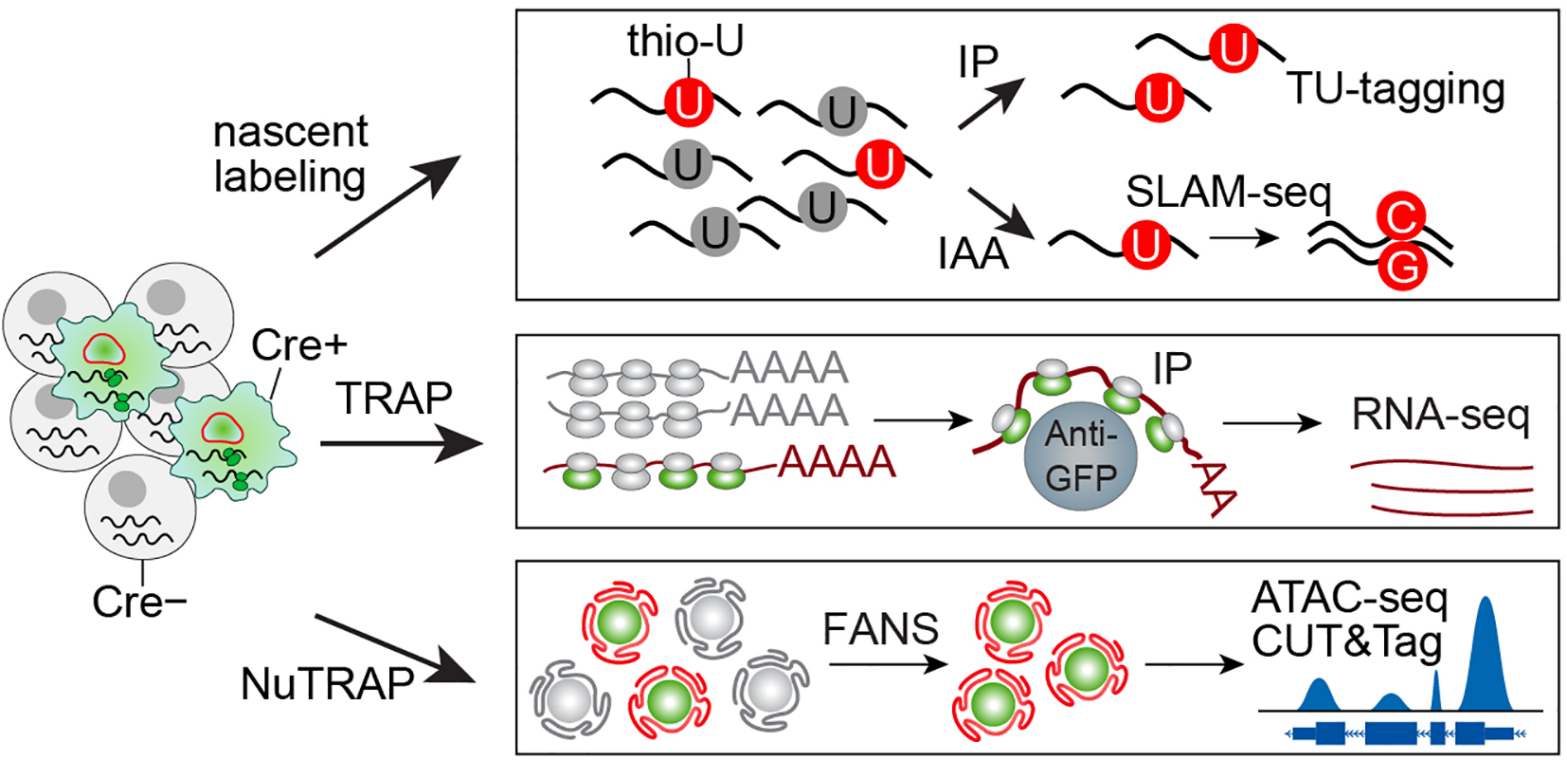
An overview of *in situ* multi-omic profiling. *In situ* profiling enables cell-specific molecular labeling through tissue-specific expression of engineered proteins. For RNA profiling, uracil phosphoribosyltransferase (UPRT) incorporates thiouracil (thio-U) into nascent RNA, which can then be enriched via either immunoprecipitation (IP) or iodoacetamide (IAA) alkylation, the latter inducing T->C and A->G mutations in thio-U-labeled transcripts. Alternatively, EGFP-tagged ribosomes allow Cre+ cell mRNA isolation through GFP immunoprecipitation. For nuclear profiling, fluorescent labeling of nuclear membrane or histone proteins permits fluorescence-activated nuclear sorting (FANS) to purify nuclei from specific cell populations, facilitating downstream epigenetic analyses.

To overcome these limitations, *in situ* methods—which analyze cells within their native tissue environment—have emerged as a powerful alternative (**Figure 2**). The core principle involves pre-labeling cellular components (e.g., nuclei, RNA, or proteins) before tissue disruption, followed by molecule-based enrichment rather than whole-cell isolation. This strategy preserves physiological context while enabling precise characterization of macrophage identity and activity.

For *in situ* nuclear profiling of specific cell populations, researchers can employ two distinct NuTRAP mouse models that fluorescently label either nuclear membranes or histones (34). These tools enable comprehensive epigenetic characterization through chromatin accessibility assays (ATAC-seq), histone modification profiling (CUT&Tag), and transcription factor binding analysis (CUT&RUN). Following rapid tissue lysis, the isolated nuclei are fixed with formalin to maintain native epigenetic states. The fluorescently labeled nuclei can then undergo fluorescence-activated cell sorting (FACS) to achieve cell-specific resolution for downstream epigenetic analyses (35).

For RNA pre-labeling, two primary methods are available: incorporation of thiouracil ribonucleotides into newly synthesized RNA or immunoprecipitation of ribosomes bound to actively translated mRNA. In the first approach, the uracil analog 4-thiouracil is converted to thio-uridine monophosphate (thio-UMP) by a parasite-derived uracil phosphoribosyltransferase (UPRT) and incorporated into nascent RNA (36). By engineering mice to express UPRT in a cell-specific manner, 4-thiouracil incorporation can be restricted to target cell populations (37). The labeled RNA can then be isolated through biotinylation or alkylation-based capture methods. The second approach is more straightforward, utilizing cell-specific expression of GFP-tagged ribosomes (translational ribosome affinity purification) (38). GFP immunoprecipitation enables enrichment of actively translated mRNAs associated with these ribosomes, providing a direct method to profile cell-specific translational activity.

Studying protein-protein interactions (PPIs) presents significant challenges, particularly *in vivo*, where many interactions are transient or weak. To address this, researchers have engineered mutant biotin ligases with rapid labeling kinetics, including TurboID, which has been adapted for use in mouse brain studies (39, 40). In this model, TurboID expression is restricted to astrocytes, enabling cell-specific biotinylation of proteins within these cells. Following tissue lysis, biotinylated proteins can be purified via immunoprecipitation for downstream analysis. Importantly, this approach can be extended to investigate macrophage specification by targeting TurboID expression to endogenous macrophage lineage factors (41), thereby enabling precise profiling of macrophage-specific PPIs in their native context.

## Discussion

Understanding macrophage plasticity and heterogeneity is essential for developing targeted therapies for diverse diseases. While the traditional M1/M2 classification has provided a foundational framework, it fails to capture the multidimensional and dynamic nature of macrophage functional states. In this mini-review, we highlight the critical importance of considering ontogenetic imprinting when interpreting macrophage functionality, as evidenced by the functional disparities between embryonic-derived and monocyte-repopulated macrophages during tissue homeostasis. Conversely, in disease contexts, particularly inflammatory conditions, niche factors drive environmental reprogramming that either directly alters tissue-resident macrophages or promotes monocyte recruitment and differentiation within inflamed tissues. Importantly, neither ontogenetic programming nor environmental cues operate in isolation; rather, their interplay shapes macrophage behavior through dynamic epigenetic mechanisms. Emerging evidence underscores the pivotal role of enhancer landscape reprogramming in this process, with characterization of these epigenetic modifications offering novel insights into macrophage regulation (42).

We propose that integrating lineage information with epigenetic profiling offers a more comprehensive approach to deciphering macrophage function. Current methods like single-cell RNA sequencing (scRNA-seq), whether performed with or without fluorescence-activated cell sorting (FACS), generate valuable data but require extensive tissue processing. These procedures disrupt the native microenvironment and induce cellular stress, potentially obscuring critical stromal influences on macrophage behavior.

To overcome these limitations, we advocate for the complementary use of emerging *in situ* techniques. These state-of-the-art tools enable macrophage investigation within their native tissue context, preserving crucial cellular interactions and microenvironmental cues. Their application promises to yield novel insights into macrophage biology while overcoming the artifacts associated with traditional isolation methods.
